# Hybrid Materials for Vascular Applications: A Preliminary In Vitro Assessment

**DOI:** 10.3390/bioengineering11050436

**Published:** 2024-04-28

**Authors:** Martina Todesco, Martina Casarin, Deborah Sandrin, Laura Astolfi, Filippo Romanato, Germana Giuggioli, Fabio Conte, Gino Gerosa, Chiara Giulia Fontanella, Andrea Bagno

**Affiliations:** 1 Department of Civil, Environmental and Architectural Engineering, University of Padua, Via Marzolo 9, 35131 Padua, Italy; 2 L.i.f.e.L.a.b. Program, Consorzio per la Ricerca Sanitaria (CORIS), Veneto Region, Via Giustiniani 2, 35128 Padova, Italy; 3 Department of Surgery, Oncology and Gastroenterology, Giustiniani 2, 35128 Padua, Italy; 4 Department of Physics and Astronomy ‘G. Galilei’, University of Padova, Via Marzolo 8, 35131 Padova, Italy; 5 Department of Neurosciences, University of Padua, Via Giustiniani, 2, 35128 Padua, Italy; 6 CNR-INFM TASC IOM National Laboratory, S.S. 14 Km 163.5, Basovizza, 34012 Trieste, Italy; 7 Department of Prevention Veterinary Services, ULSS 3 Serenissima, P.le S.L Giustiniani 11/D Mestre, 30174 Venice, Italy; 8 Department of Cardiac, Thoracic Vascular Sciences and Public Health, University of Padova, Via Giustiniani 2, 35128 Padova, Italy; 9 Department of Industrial Engineering, University of Padua, Via Marzolo 9, 35131 Padova, Italy

**Keywords:** hybrid materials, vascular graft, tissue engineering, regenerative medicine, decellularized pericardium

## Abstract

The production of biomedical devices able to appropriately interact with the biological environment is still a great challenge. Synthetic materials are often employed, but they fail to replicate the biological and functional properties of native tissues, leading to a variety of adverse effects. Several commercial products are based on chemically treated xenogeneic tissues: their principal drawback is due to weak mechanical stability and low durability. Recently, decellularization has been proposed to bypass the drawbacks of both synthetic and biological materials. Acellular materials can integrate with host tissues avoiding/mitigating any foreign body response, but they often lack sufficient patency and impermeability. The present paper investigates an innovative approach to the realization of hybrid materials that combine decellularized bovine pericardium with polycarbonate urethanes. These hybrid materials benefit from the superior biocompatibility of the biological tissue and the mechanical properties of the synthetic polymers. They were assessed from physicochemical, structural, mechanical, and biological points of view; their ability to promote cell growth was also investigated. The decellularized pericardium and the polymer appeared to well adhere to each other, and the two sides were distinguishable. The maximum elongation of hybrid materials was mainly affected by the pericardium, which allows for lower elongation than the polymer; this latter, in turn, influenced the maximum strength achieved. The results confirmed the promising features of hybrid materials for the production of vascular grafts able to be repopulated by circulating cells, thus, improving blood compatibility.

## 1. Introduction

Hybrid materials can be considered a new frontier of biomaterial science. They represent an innovative field of study, particularly conceived for tissue-engineering purposes toward the production of materials that can really integrate with the host organism. According to the definition given by Casarin et al. [1], hybrid materials are a group of composite materials made by combining biological tissues with synthetic polymers, thus merging the structural, chemical, and physical properties of both types of materials, which remain separate on a microscopic scale [1,2,3]. Hybrid materials have been applied in various areas of regenerative medicine and can be made by coupling organic–inorganic or organic–organic substances [4]. Hybrid materials are expected to provide new properties in addition to the specific characteristics of individual components. Biological materials provide biocompatibility and act as a scaffold to support the growth of patient cells due to the presence of extracellular matrix growth factors; synthetic materials can improve mechanical patency and durability [5,6]. Therefore, hybrid materials represent an optimal compromise between stability, mechanical properties, and biocompatibility [5].

Hybrid materials have been exploited in the orthopedic field where, for example, a hybrid material was made by coupling hydroxyapatite and chitosan; in the dermatological field where polycaprolactone/TiO_2_ coated type I collagen was used for skin regeneration [7]; in the urological field where collagen was coupled with another synthetic material (e.g., poly[lactic acid-co-caprolactone], poly lactic glycolic acid or poly lactic glycolic acid) [8,9,10]. On the other hand, biological tissues are often used for cardiovascular applications, both decellularized or chemically fixed, and coupled with polymers, e.g., polycaprolactone or polycarbonate urethane [11,12,13,14].

The work presented herewith regards the cardiovascular field, and it is aimed at creating a bioengineered solution for the replacement of damaged vascular tissues to face some of the most common cardiovascular diseases (CVDs). CVDs are a group of disorders that affect the heart and blood vessels and, in agreement with the World Health Organization (WHO), account for 17.9 million deaths per year, representing 32% of all deaths. They can be treated with surgical procedures such as angioplasty, stent insertion, or atherectomy; a vascular graft can be used to replace or bypass a damaged or obstructed vessel [15]. An ideal therapy requires the replacement of the injured vessel with an autologous one. The saphenous vein and the internal thoracic artery represent the gold standard grafts for small vessel diameters; they have excellent patency [16,17], but present some disadvantages. In particular, they require invasive harvesting procedures; are frequently deemed unsuitable due to previous pathologies, which can also impact the mechanical properties of tissues [18,19]; and exhibit failure rates of approximately 50% within a decade [15,17,20].

Synthetic vascular grafts, such as those made of expanded polytetrafluoroethylene (ePTFE) or polyethylene terephthalate (PET), are employed to fulfill the shortage of autologous substitutes. They possess good features in terms of impermeability, flexibility, and compliance, but exhibit long-term risk of infection, inflammation, thrombogenicity, and intimal hyperplasia formation [21]. In particular, hyperplasia occurs near anastomoses, and it is caused by a variety of circumstances, including hemodynamic factors that alter blood flow, compliance and vascular diameter mismatch, injury to or lack of endothelial cells, suture line stress concentration, and trauma during surgery [22,23,24,25]. Additionally, synthetic grafts have fair patency rates, especially when used to replace small-diameter vessels; for example, PTFE grafts used for coronary artery bypass have a patency of 20% [15], resulting in unacceptable long-term outcomes and a chronic risk of infection [21]. Moreover, a further critical aspect of synthetic cardiovascular grafts is the need for sustained anticoagulant therapy; it interferes with the normal hemostatic process and can convert clinically insignificant bleeding to clinically significant [26,27].

In the search for the optimal material to produce engineered vascular substitutes, the following characteristics can be listed. First, biocompatibility: the graft has to be noncytotoxic to prevent any adverse immune reaction, i.e., chronic inflammation, initiation of the complement cascade, and activation of the adaptive immune system. It also needs to be porous enough to let nutrients diffuse to cells and eventually degrade without toxic byproducts, with a degradation rate that enables tissue regeneration [28]. A vascular graft of engineered tissue should also be suitable for implantation, with kink resistance, and be suitable for easy handling and suturing. The graft is expected to ultimately favor the healing process after surgery and integrate with the host tissue.

In order to create vascular substitutes with the necessary technological qualities and appropriate physicochemical behavior, hybrid materials have been proposed [29,30,31], which, as previously reported, have been developed to combine mechanical and biological properties as closely as possible to those of native tissues, while improving functional characteristics such as impermeability [1,32,33].

In the present study, bovine pericardium has been decellularized and coupled with two commercial polymeric formulations, Chronoflex AR and Chronoflex ARLT, to develop innovative materials that are able to effectively integrate with the patient’s body; in particular, the decellularized tissue mimics native vascular tissue and encourages cell migration and proliferation; the polymeric material ensures impermeability and mechanical resistance. The selected polymers are biocompatible thermoplastic polycarbonate urethanes, which are widely used in the cardiovascular field due to their excellent blood compatibility [34,35]. They are also resistant to biologically induced environmental-stress-cracking (BI-ESC) phenomena, which weaken the mechanical integrity of the implant and cause surface fissures from which further deterioration may occur [28]. On the other hand, bovine pericardium has been frequently used in several biomedical applications (e.g., prosthetic heart valves and vascular grafts) but only after glutaraldehyde fixation, which can result in cytotoxic effects and induce structural malfunction as a consequence of tissue degradation due to calcification [36]. To prevent these drawbacks, biological tissue has been decellularized, while maintaining its native physical, chemical, and mechanical properties [37]. In other studies, the decellularized bovine pericardium was used and functionalized with the REDV peptide to improve cell growth and migration [38].

In the present work, the hybrid materials have been physiochemically assessed and tested in vitro in direct contact with human umbilical vein endothelial cells (HUVECs). Mechanical tests have been also performed to characterize the behavior of the hybrid materials and to evaluate the effect of the tensile loading cycle.

## 2. Materials and Methods

### 2.1. Pericardia Preparation

Fresh bovine pericardium from healthy animals (Holstein Friesian calves, 7 months old, weighing between 300 and 350 kg) was obtained from local slaughterhouses, and treated within 3 h after sacrifice. The Italian government monitored the slaughterhouses’ conformity to the EC laws 1099/2009 on animal welfare and protection, and the corresponding legal authority for animal welfare (Food and Consumer Product Safety Authority) gave the approval. Each pericardium was separated from its attachment at the base of the heart, surrounding the major vessels, and then brought to the laboratory in a cold saline solution (0.9% *w*/*v* NaCl, Sigma-Aldrich, St. Louis, MO, USA). The anterior left ventricular portion of the bovine pericardium was cut into rectangular specimens (80 × 80 mm^2^) and the adhering fatty tissue was gently removed [37,39]. Tissue samples were decellularized following the Tergitol procedure [37]. Briefly, tissues were protected from lytic processes by a protease inhibitor solution followed by a hypo/hypertonic solution alternated with Tergitol (1–0.1%/v) and 10 mM of an anionic surfactant; sodium cholate was used to extract cellular components. All extractions were carried out in a solution containing 10 mM sodium ascorbate and 5 mM EDTA that was kept under a nitrogen atmosphere to prevent oxidation. After washing with saline solution, 10% isopropanol was used. Finally, tissues were exposed to an aspecific endonuclease (Benzonase™) in equilibration buffer (50 mM Tris-HCl, 1 mM MgCl_2_) at 37 °C for 48 h to fragment double- and single-strand nucleic acids. All reagents have been purchased from Sigma-Aldrich.

### 2.2. Hybrid Membrane Fabrication

Hybrid materials were produced by solution casting and solvent evaporation as previously described by our group [29,40]. Decellularized bovine pericardium (DBP) samples were placed on a custom-made aluminum frame and a thin layer of polycarbonate urethane (PCU) was poured on the fibrosa side of the tissue; thereafter, samples were treated in a vacuum oven under aspiration for 24 h at 40 °C (Raypa, Barcelona, Spain). Two polycarbonate urethane solutions at 22% *v*/*v* in dimethylacetamide (DMAc) supplied by AdvancSource Biomaterials (Wilmington, MA, USA) were used. Two hybrid materials were realized: decellularized bovine pericardium coupled with Chronoflex AR (DBP AR) and decellularized bovine pericardium with Chronoflex ARLT (DBP ARLT).

### 2.3. Tissue Sterilization

Sterilization was carried out according to Fidalgo et al. [41]. Briefly, hybrid materials were placed at 37 °C for 24 h under constant agitation in a solution with antibiotics and antimycotics (AA) that contained vancomycin hydrochloride (50 mg/L, Sigma Aldrich), gentamicin (8 mg/L, Sigma Aldrich), cefoxitin (240 mg/L, Sigma Aldrich), and amphotericin B (25 mg/L, Carlo Erba, Cornaredo, Italy). Then, hybrid materials were washed with PBS and treated with 0.1% *v*/*v* peracetic acid (PAA) solution for 3 h at room temperature. Samples received a final 1-h PBS washing to remove all remaining chemicals and cells.

### 2.4. ATR-FTIR Analysis

The Nicolet iS-50 spectrometer (Thermo Fisher Scientific, Waltham, MA, USA) with a single-reflection diamond/ZnSe crystal ATR attachment was used to investigate the chemical composition of the hybrid materials surfaces of both the polymeric and pericardial sides. In particular, the spectra obtained from the pericardial side were compared to those from DBP alone to reveal the possible presence of polymer and/or possible denaturation of typical ECM proteins.

Hybrid materials were cut into 10 × 10 mm^2^ square samples (n = 3 for each group) and equilibrated in deuterium oxide, supplied by Janssen (Beerse, Belgium) in order to reduce the interference of water bands in the amide-I region typical of the pericardial tissue [42]. The instrument’s crystal surface was covered with material patches, and proper contact between the material and the crystal was ensured by using the ATR’s pressure arm. All analyses were performed at room temperature. Using 64 scans each, the transmittance of the sample and the background were acquired, and infrared spectra were collected between 4000 and 500 cm^−1^. Spectra were analyzed using a Matlab^®^ R2021a script (Mathworks, Natick, MA, USA) [43].

### 2.5. Two-Photon Microscopy

Two-photon microscopy was performed to evaluate the DBP AR and DBP ARLT; in particular, the pericardial structure was assessed to understand if and how much the polymer penetrates the tissue. DBP AR and DBP ARLT samples (n = 3 each) were rinsed in PBS for 24 h, then fixed at room temperature in PFA (Bioptica, Milan, Italy) for 20 min and subsequently stored in PBS (Sigma Aldrich, St. Louis, MO, USA).

The images were obtained using a custom multiphoton microscope developed by Filippi et al. [44]. Images were acquired with a fixed magnification using an Olympus 25X immersion objective (Tokyo, Japan) with a numerical opening of 1.05 (1024 × 1024 pixels), an average signal over 70 consecutive frames, with a pixel size of 0.4 μm. Analysis was performed by measuring the second harmonic generation (SHG) to reveal collagen structure, while the blue channel signal was acquired to reveal the presence of the polymers. Each hybrid material sample was then excited by an 800 nm laser, and the detector recorded the light that was released in two channels: one at 400 nm, which corresponds to the formation of the second harmonic and where only collagen is detectable, while the polymer was found in the second channel, which operates between 435 and 500 nm (blue channel).

DBP, DBP AR, and DBP ARLT were studied using the same parameters to compare them. Several z-stacks were obtained and the RAW images were elaborated using ImageJ version 1.53o, an open-source software (NIH, Bethesda, MD, USA) [45]. For each sample, three regions were analyzed and SHG and blue intensity data were acquired at different depths from the surface of the pericardial side up to 242 μm. The two signals were normalized and overlapped to reveal how deeply polymers penetrate the decellularized tissue.

By using the plug-in OrientationJ version 2.0.5, which involves the use of the Fast Fourier Transform to analyze collagen distribution, two parameters were estimated: SHG coherency and SHG intensity [37,46,47,48]. The SHG coherency values account for the organization and distribution of collagen fibers. A value of 1 indicates that the fibers are strongly orientated (anisotropy), while a value of 0 indicates that there is no orientation (isotropy). With regard to the SHG intensity parameter, a high value corresponds to a high concentration of collagen.

The *t*-tests were performed to statistically compare the values of intensity and coherency of DBP AR and DBP ARLT with DBP as a reference group.

### 2.6. Mechanical Characterization

All materials were cut into dog-bone-shaped specimens by means of a homemade cutter with a gauge length of 5 mm and width of 2 mm; specimens were cut along the predominant direction of the collagen fibers.

The thickness of the DBP AR and DBP ARLT samples was measured directly using a Mitutoyo digital caliber (model ID-C112XB, Mitutoyo America Co., Aurora, IL, USA), while the thickness of the DBP samples was measured by sandwiching them between two glass slides whose thickness was then subtracted.

A tensile testing machine (TRAMA, IRS, Padova, Italy) was used for the mechanical characterization of all samples. It is equipped with four linear actuators and 50 N loading cells and is operated by a dedicated LabVIEW 2019 software (National Instruments, Austin, TX, USA).

The response to the load of DBP and hybrid materials was assessed by the failure test. Each sample was preloaded to a maximum of 0.1 N before being extended until failure at a rate of 4% s^−1^. Samples were continuously wetted with a saline solution to prevent dehydration. From the stress-strain curves, failure strain (FS), ultimate tensile strength (UTS), and Young’s modulus were calculated for each sample [37]. Since the stress-strain curve of soft tissues has a typical J shape, it can be divided into two regions that are characterized by different stiffness. The first part of the curve is usually termed the elastin phase or toe region, while the second is called the collagen phase or linear region, to indicate the main contribution of collagen to the mechanical response to load. For this reason, two distinct Young’s moduli were calculated to better characterize the materials: E_1_ was calculated as the slope of the toe region between 1% and 10% deformation, and E_2_ was calculated as the slope of the linear portion of the curve just before sample rupture.

To investigate the effect of loading cycles on material resistance, a fatigue test was conducted. After the application of loading cycles up to 20% deformation for 3600 s with a strain rate of 26% s^−1^, each specimen was loaded until rupture. Tests were performed at room temperature and samples were immersed in a saline solution (0.9% NaCl) to prevent dehydration. The FS and UTS of the fatigue stress tests were calculated and compared with the same parameters obtained from the failure tests.

All parameters were calculated by means of an in-house developed Matlab^®^ R2021a script and statistical analysis was performed with the *t*-test (GraphPad Prism 10 Software, San Diego, CA, USA). Significance was set at *p* < 0.05.

### 2.7. Sterility Test

The European Pharmacopoeia 2.6.1 for biological samples was followed to assess the efficacy of sterilization treatments [49]. Hybrid materials (DBP AR and DBP ARLT) and polymers (CF AR and CF ARLT) were cut with an 8 mm puncher; n = 2 samples for each group were tested. Specimens were placed in 4 mL sterile tubes with bacteria-specific media, i.e., Soybean Casein Digest Medium (Cat no. 22092) and Fluid Thioglycollate Medium (Cat no. T9032), both supplied by Sigma Aldrich in powder and reconstituted by adding Milli-Q water. The Soyabean Casein Digest Medium tubes were incubated at room temperature, while the Fluid Thioglycollate Medium tubes were maintained at 35 °C; turbidity and color of both solutions were checked every 24 h for 14 days. Only-medium tubes were considered as negative controls. In the thioglycollate medium, any change in color or turbidity indicates the presence of either aerobic or anaerobic bacteria; in Soybean Casein Digest Medium, it indicates the presence of fungi and aerobic bacteria.

### 2.8. Cytotoxicity Assay

The in vitro direct contact cytotoxicity test for DBP, DBP AR, DBP ARLT, CF AR, and CF ARLT was validated following the ISO 10993-5 [50]. Human Umbilical Vein Endothelial Cells (HUVECs) were from a single donor (cod. C-12200). Cells were expanded in Endothelial Cell Basal Medium 2 (cod. C-22211) containing Endothelial Cell Growth Medium 2 Supplement Pack (cod. C-39211) at 37 °C in a 5% CO_2_ incubator with a humidified atmosphere. HUVECs and media were provided by PromoCell (Heidelberg, Germany).

Hybrid materials (DBP AR and DBP ARLT) and polymers (CF AR and CF ARLT) were cut with an 8 mm diameter puncher, while DBP was cut in squared samples (20 × 20 mm^2^) and fixed in a custom-made insert with the same dimensions. Specimens were put in a 24-well plate in aseptic conditions. Before being seeded with cells, the materials were soaked in a fresh culture medium overnight. All samples had a culture area of 0.5 cm^2^. The HUVECs were seeded onto each material, at passage 4, with a density of 30,000 cells/cm^2^ and kept on the scaffolds for 7 days. Samples were collected and analyzed on day 1 and day 7.

Metabolic activity and cell viability were assessed by selective reduction of tetrazolium salt by means of the WST-1 test (Boster Biological Technology, Pleasanton, CA, USA). Tissue samples were incubated with a WST solution 2.5% (*v*/*v*) in a culture medium at 37 °C under 5% CO_2_ for 1.5 h. The amount of reduced WST-tetrazolium was quantified by absorption at 450 nm in a culture medium with Spark 10M TECAN (Tecan, Männedorf, Switzerland).

Cellular viability was investigated by means of the LIVE/DEAD assay. At each time point, seeded samples were incubated with a culture medium added with Calcein AM (2 µM) and ethidium homodimer-1 (4 µM) provided by the LIVE/DEAD staining kit (Invitrogen, Carlsbad, CA, USA) to highlight live and dead cells, respectively in green and red stain. Seeded tissues were incubated at 37 °C and 5% of CO_2_ for 45 min. At the end of the incubation period, the staining solution was replaced with a fresh medium.

Direct immunofluorescence staining was performed for all sample materials to evaluate cell distribution and organization. Samples were fixed with 4% (*w*/*v*) formaldehyde for 20 min and then incubated with 0.1% Triton X-100 solution for 15 min, after 2 washes in PBS. Samples were incubated with 1% (*w*/*v*) bovine serum albumin and F-actin was counterstained by using Phalloidin (1:200, 65906, Sigma-Aldrich) while the nuclei were stained with 4′,6-diamidino-2-phenylindole (DAPI, Invitrogen), following producer’s instructions.

LIVE/DEAD and immunofluorescence images of samples were acquired with a Leica AF6000 epifluorescence microscope, connected to a Leica DC300 digital camera and equipped with LAS AF 4.0 software (Leica Micro-System, Wetzlar, Germany). Image processing was conducted with Fiji software version 2.9.0/1.53.

## 3. Results

### 3.1. Membrane Aspect and Polymer Penetration

Four materials were produced and characterized: two hybrid materials, which were obtained by combining CF AR and CF ARLT with decellularized bovine pericardium, and two polymeric membranes made with CF AR and CF ARLT alone, which were used as a control.

**Figure 1:**
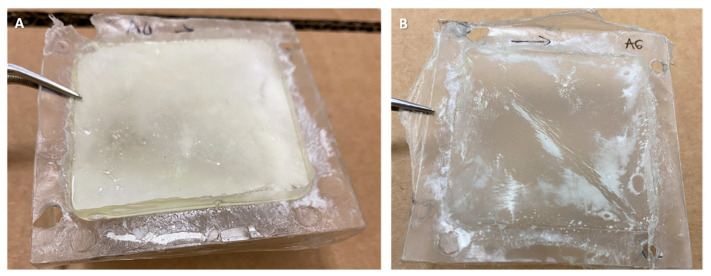
Hybrid materials (50 × 50 mm^2^) produced by coupling the decellularized bovine pericardium with Chronoflex AR (**A**) and Chronoflex ARLT (**B**). The two polymer formulations have the same chemical composition, but Chronoflex ARLT is added with 9% microsilica, which makes it less sticky.

Hybrid materials were produced as 2D membranes (Figure 1), with a thickness of 0.58 ± 0.08 mm for DBP AR and 0.60 ± 0.12 mm for DBP ARLT, the thickness of the membrane made with CF AR and CFARLT is 0.68 ± 0.46 mm and 0.93 ± 0.54 mm, respectively, and DBP results have a thickness of 0.29 ± 0.06 (Table 1).

**Figure 2:**
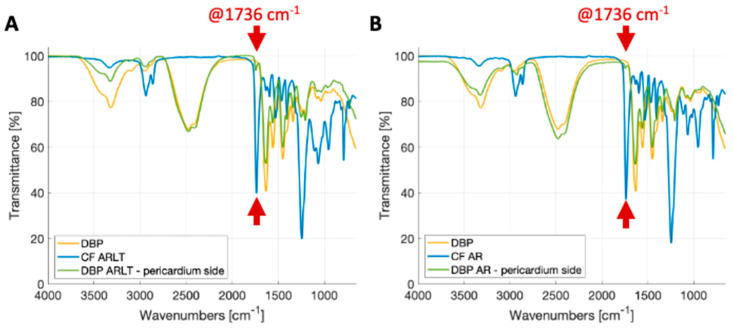
FTIR-ATR spectra: (**A**) Chronoflex ARLT (blue line), bovine pericardium (yellow line) and DBP ARLT (green line); (**B**) Chronoflex AR (blue line), bovine pericardium (yellow line) and DBP AR (green line). Red arrows highlight the 1736 cm^−1^ wavelength that is typical of the polycarbonate urethane group.

Figure 2 shows the FTIR-ATR spectra for DBP, polymers (CF AR and CF ARLT), and hybrid materials (DBP AR and DBP AR-LT) from the pericardial side. Spectra were collected in the frequency range of 4000–500 cm^−1^ and are largely overlapped. This indicates that the structure of the pericardial ECM proteins has not significantly changed. Notably, in the signal acquired from the pericardial side of hybrid materials, characteristic collagen peaks emerged at approximately 1650 cm^−1^ and 1560 cm^−1^, corresponding to the amide I and amide II bands, respectively. The amide I peak primarily corresponds to the stretching of the C=O group, while the amide II peak is attributed to the stretching of C–N and the bending of the N–H group [51,52,53]. The amide III peak, caused by N–H bending, is centered at ~1245 cm^−1^, while the CH and COH transmittance peaks of carbohydrates, which are the main constituents of glycosaminoglycans (GAGs), are typically found between ~1250 and ~1000 cm^−1^ [54].

As to the spectra of the commercial polymers, such as CF AR and ARLT (Figure 2A,B), the two signals exhibit common peaks in ~1737 cm^−1^ and ~1251 cm^−1^, which are due to urethane (C=O urethan amide I bond) and O–C–O bonds in carbonate groups, respectively [28]. The reduction in signal transmittance for Chronoflex ARLT between 1200–900 cm^−1^ is due to the presence of silica microparticles.

Regarding the spectra acquired on the pericardium side of hybrid materials, a transmittance peak can be observed at approximately 1737 cm^−1^. It is characteristic of the urethane group and can be assigned to the presence of polymer in traces after diffusion through the biological tissue. This peak is more pronounced in the DPP ARLT membrane than in the DPP AR one. This is probably due to the different diffusion capacities of the two polymeric formulations. Moreover, a decrease in transmittance for DBP AR and DBP ARLT compared with DBP also indicates the presence of polymer traces on the pericardial side.

It is likely to note that variations in the spectra of the investigated materials are present: they can be simply due to artifacts or, more probably, to changes in the chemical compositions of the samples as in the case of biological materials due to inter-individual variations. Therefore, it is of major interest to look at the presence of the peaks more than their intensity.

**Figure 3:**
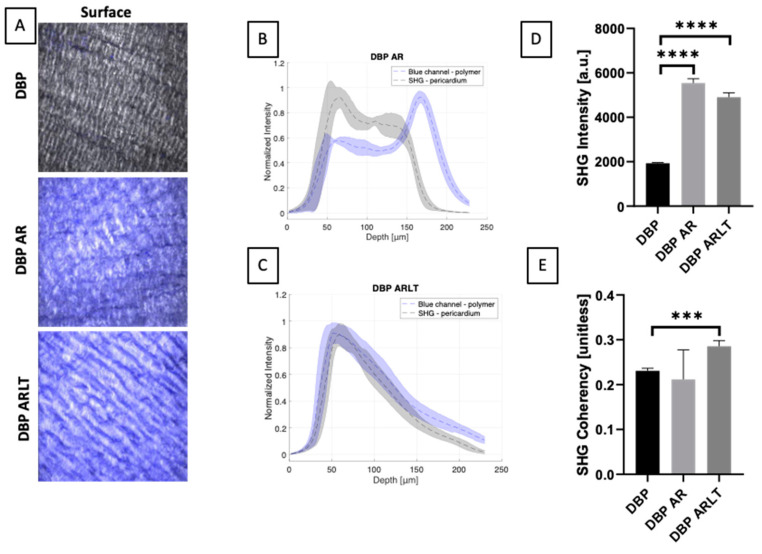
Two-photon microscopy images were acquired on the surfaces of DBP from the serosa side and on hybrid materials surfaces (DBP AR and DBP ARLT). Merge Z-stack of SHG and blue channel signals are reported (**A**). Graphs of normalized intensities of SHG and blue channel for DBP AR (**B**) and DBP ARLT (**C**) are reported (mean ± SD). The SHG intensity (**D**) and coherency (**E**) values were calculated from two-photon microscopy images of the pericardium surface. The *t*-test was applied to compare DBP with DBP AR and DBP ARLT, respectively, and a significant variation was reported for the SHG intensity of DBP AR and DBP ARLT compared with DBP, while the SHG coherency values of DBP ARLT are significantly different if compared with DBP. Data in the histograms are presented as mean ± SD. *** *p* < 0.001, **** *p* < 0.0001.

Two-photon microscopy enables high-resolution imaging of biological tissues, highlighting the presence of collagen fibers (as indicated by the SHG signal). The resulting images (Figure 3A) provide valuable information on tissue structure, composition, and organization. Measurements of coherency and intensity parameters allowed quantifying the amount of collagen and its overall organization. Regarding the fibrillar structure of the pericardial surface, collagen typically exhibits densely packed fibrils, providing structural support and strength to the tissue. Collagen fibrils show a certain degree of organization in terms of orientation. The coherency parameter shows a significant difference (*p* = 0.0002) between DBP and DBP ARLT. Moreover, collagen density was assessed by the intensity parameter, which shows a significant difference between DBP and DBP AR (*p* < 0.0001) and between DBP and DBP ARLT (*p* < 0.0001) (Figure 3D,E).

From the images of DBP AR and DBP ARLT, it is also possible to observe the presence of the signal in the blue channel on the pericardial surface (Figure 3B,C). This confirms the presence of polymer traces.

### 3.2. Mechanical Characterization

**Figure 4:**
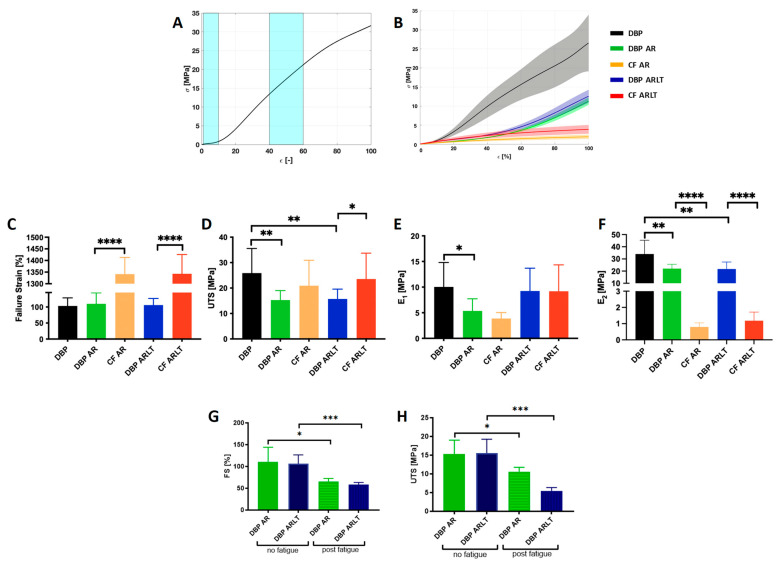
Results of uniaxial tensile tests (**A**–**F**) and fatigue tests (**G**,**H**). (**A**) typical stress-strain curve of soft tissue: in cyan boxes, the deformation ranges where modulus E_1_ and modulus E_2_ are calculated. (**B**) Stress-strain curve obtained from tensile test. Hybrid materials do not show significant differences in FS (**C**) of DBP and DBP AR if compared with DBP, while they exhibit significant differences as to the UTS values, which are lower than those of DBP (**D**). E_1_ shows a significant difference between DBP and DBP AR (**E**); E_2_ values of both DBP AR and DBP ARLT are significantly lower than DBP, but significantly higher than CF AR and CF ARLT, respectively (**F**). Fatigue cycle tests show that both UTS and FS values decrease after loading cycles (**G**,**H**): the decrease is greater for DBP ARLT than for DBP AR. Data on histograms are expressed as mean ± SD. * *p* < 0.05, ** *p* < 0.01, *** *p* < 0.001, **** *p* < 0.0001.

The results of mechanical characterization are shown in Figure 4. A typical stress-strain curve for a soft tissue (A) and the response to the load of the investigated materials (B) are depicted. As for the failure test, from the graphs (Figure 4C) it is likely that the FS achieved by the hybrid materials (109.9 ± 34.16% for DBP AR and 106 ± 20.69% for DBP ARLT) does not differ significantly from that of DBP (103.3 ± 25.32). On the other hand, there is a significant difference (*p* < 0.0001) in the FS values when comparing hybrid materials and both polymers, i.e., CF AR and CF ARLT (1341 ± 71.7% and 1343 ± 82.78%, respectively).

Furthermore, the UTS of the hybrid materials (Figure 4D), which reached 15.28 ± 3.72 MPa for DBP AR and 15.72 ± 3.85 MPa for DBP ARLT, shows significant differences compared with DBP, whose UTS is 25.9 ± 9.66 MPa (*p* = 0.0046 for DBP AR; *p* = 0.0063 for DBP ARLT). Moreover, the UTS value achieved by DBP ARLT is statistically different from that of CF ARLT (*p* = 0.0465).

To check the stiffness of the hybrid materials under investigation, two moduli were calculated from the stress-strain curves. The E_1_ (Figure 4E) shows a significant difference between DBP and DBP AR (*p* = 0.0117), E_1_ = 10.04 ± 4.78 MPa, and E_1_ = 5.35 ± 2.37 MPa, respectively; there is no significant difference between DBP AR and DBP ARLT, and CF AR and CF ARLT, respectively. The E_2_ (Figure 4F) shows a significant difference between the value reached by DBP compared with DBP AR (*p* = 0.0057) and DBP ARLT (*p* = 0.0061), while an E_2_ value of 34.01 ± 11.32 MPa was calculated for DBP; DBP AR and DBP ARLT reached 22.09 ± 3.51 MPa and 21.74 ± 5.71 MPa. There is also a significant difference between DBP AR and CF AR (*p* < 0.0001) and DBP ARLT and CF ARLT (*p* < 0.0001). The two polymers, CF AR and CF ARLT, have E_2_ values of 0.79 ± 0.24 MPa and 1.18 ± 0.52 MPa, respectively.

The fatigue test allowed the investigation of the effects of deformation cycles applied to the materials before elongating them to failure. The results are shown in Figure 4G,H. There is a significant decrease in the strain and strength parameters achieved by the hybrid materials subjected to cyclic stress. As for FS, the decrease is 40.79% for DBP AR (*p* = 0.0133) and 44.8% for DBP ARLT (*p* = 0.0007). A decrease appears for UTS, and it was found to be 30.62% for DBP AR (*p* = 0.020) and 64.75% for DBP ARLT (*p* = 0.0003).

### 3.3. Materials Sterilization and Cytotoxicity

The sterilization of materials was carried out according to European Pharmacopoeia 2.6.1 for biological samples. Samples of each material were immersed in two media (Soybean Casein Digest Medium and Fluid Thioglycollate Medium) to reveal the presence of aerobic and anaerobic bacteria and fungi due to possible contamination. No change in media turbidity and color was found by visual inspection, confirming the sterilization effectiveness.

The cytotoxicity of hybrid materials was tested following ISO 10993-5 [50] by directly seeding cells on the serosal side of both the pericardia alone (used as controls) and the hybrid materials as well as on both polymers (used as controls). Cell growth and viability were quantitatively assessed by WST-1 assay and qualitatively by live and dead staining and immunofluorescence 1 and 7 days after seeding.

**Figure 5:**
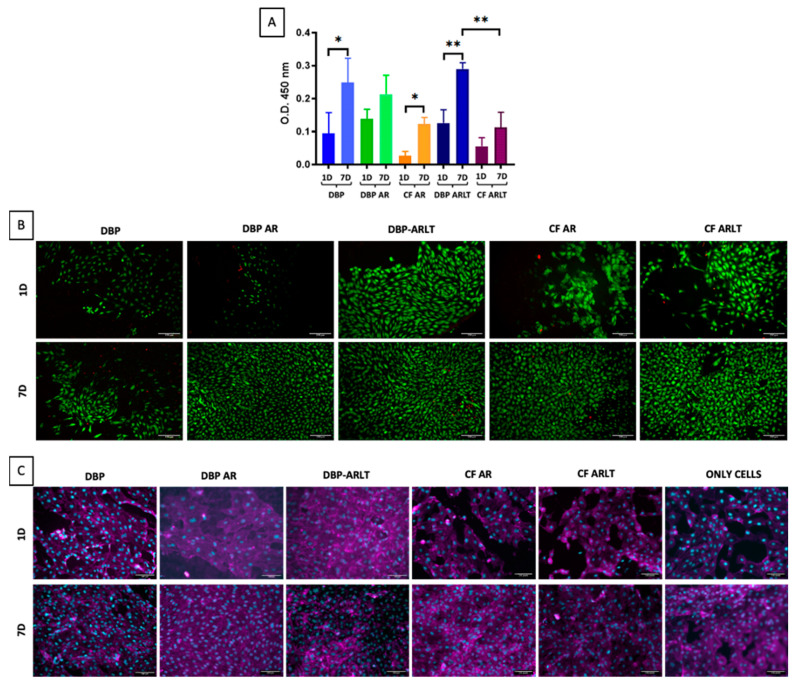
(**A**) Cytotoxicity assessment: optical density data from the WST test on seeded materials (30,000 cells/cm^2^). (**B**) Live/dead staining: cell proliferation in DBP, DBP AR, CF AR, DBP ARLT, and CF ARLT (rows) on day 1 (1D) and day 7 (7D). Calcein AM (green) is used to stain live cells and ethidium homodimer-1 (red) is used to stain dead cells. (**C**) Phalloidin (magenta) and DAPI (cyan) immunofluorescence staining show cells seeded over DBP, DBP AR, CF AR, DBP ARLT, and CF ARLT (rows) on day 1 (1D) and day 7 (7D); cells on the plastic control surface are presented in the last column. Images were acquired at 20x magnification, * *p* < 0.05, ** *p* < 0.01.

After 7 days, all tested materials were able to enhance cell proliferation, according to the WST-1 data (Figure 5A). When comparing the values obtained after 1 day with those obtained after 7 days, the optical density (O.D.) acquired at 450 nm, which is directly related to cell viability, is significantly higher for DBP (*p* = 0.0238), CF AR (*p* = 0.0275), and DBP ARLT (*p* = 0.003). Regarding DBP AR and CF ARLT, there is a slight but noticeable increase in cell viability after 7 days. Furthermore, 7 days after cell seeding, there is a significant difference in cell viability for DBP ARLT and CF ARLT (*p* = 0.0079), but not between DBP and DBP AR or DBP and DBP ARLT. Analysis of HUVECs proliferation (Figure 5B) in DBP, DBP AR, CF AR, DBP ARLT, and CF ARLT (columns) on day 1 and day 7 (row) was carried out with live/dead staining. Calcein AM (green) is used to stain live cells, while ethidium homodimer-1 (red) is used to stain dead cells. From the images, it is possible to recognize that the HUVECs have a round morphology, cell proliferation increases after 7 days, and there are no dead cells. The immunofluorescence staining (Figure 5C), showing the cytoskeleton F-actin (in magenta) and cell nuclei (in blue), reveals that cells are evenly distributed on the materials.

## 4. Discussion

The heart is enveloped by a tough double-layered membrane called pericardium. Animal pericardium can be used as a biomaterial suitable for several clinical purposes. Typical applications of bovine pericardium include tissue and artery repair, bioprosthetic heart valves, and cardiovascular device production. Numerous studies have shown that pericardium, compared to other prosthetic patches, is more biocompatible, easier to handle, less prone to suture line bleeding, and even less prone to infection [55].

The new frontier of tissue engineering for regenerative medicine is the creation of constructs able to perfectly integrate with the host organism. To this aim, decellularization of animal tissues is a promising approach. Thanks to decellularization, it is possible to eliminate immunogenic components from animal tissues and produce scaffolds that can accommodate the patient’s own cells since they possess an adequate structure of extracellular matrix with growth factors that influence cell mitogenesis, chemotaxis, and differentiation [56]. Nevertheless, decellularized tissues can occasionally lose patency and impermeability together with the mechanical strength of native biological tissues. Hybrid materials, which are obtained by combining biological and synthetic materials, represent a promising solution to join the biocompatibility of the former with the mechanical features of the latter [1]. Therefore, the aim of this study was to fabricate and preliminarily analyze a material intended to overcome the disadvantages associated with the grafts currently utilized in clinical applications, such as polyethylene terephthalate and polytetrafluoroethylene. These grafts are employed to address the absence of autologous tissues (saphenous vein, internal mammary artery, and radial artery), which may not always be readily available due to patient-specific conditions [57]. However, synthetic materials often exhibit compliance mismatches with native vessels and possess thrombogenic surfaces, effects that are enhanced under low-flow conditions. In this study, the aim was to fabricate a material by coupling a polycarbonate urethane, with a natural scaffold derived from decellularized pericardium.

The polymeric materials used are commercially available in two formulations: Chronoflex AR and Chronoflex ARLT. Indeed, Chronoflex ARLT contains 9% fumed silica, which makes it less tacky [29]. Decellularized pericardium was cultured under conditions optimized for cell proliferation, and thanks to the presence of extracellular matrix protein, the regeneration of tissue-like endothelium was promoted.

Hybrid materials were realized by the solution casting technique. The decellularized pericardium was coated with the polymer in solution and the solvent was subsequently evaporated. The hybrid materials were chemically and physically analyzed, and their mechanical properties were assessed. Macroscopically, the decellularized pericardium and the polymer appeared to well adhere to each other, and the two sides are clearly distinguishable. From FTIR analysis, which provides the chemical fingerprint of the material, since it is rich in information on the structure of the functional groups of the sample analyzed, it was possible to estimate the degree of penetration of the polymer within the decellularized tissue [58]. The chemical composition of the pericardium remained almost unchanged when compared with the decellularized pericardium alone; however, there are traces of polymer penetrating the tissue during membrane fabrication. This result is confirmed by the images acquired by two-photon microscopy, which provides quantitative and qualitative analyses, confirming that in the DBP ARLT membrane, the polymer penetrates more deeply than in the DBP AR membrane. This analysis also allowed assessing the structure of the pericardium, which retains the typical wavy conformation of collagen bundles when compared with the collagen structure in DBP alone. However, the intensity value, which is directly proportional to the amount of collagen, of DBP AR and DBP ARLT, is significantly higher than that of DBP. This evidence can be explained because the biological tissue is dried during the preparation of the membranes. This causes a higher packing of collagen fibers, resulting in an increased intensity value, which also results in an increased coherency value. An exhaustive structural characterization of pericardial samples by means of two-photon microscopy and histological analysis has been proposed in [37].

Concerning the mechanical resistance, the maximum elongation of DBP AR and DBP ARLT is mainly affected by the pericardium, which allows for lower elongation than the polymer; in contrast, the maximum strength achieved by the hybrid materials is influenced by the polymer. The stiffness was calculated in two regions of the stress-strain curves: in the initial part, where the un-crimped fibers are loaded, and in the region where the aligned collagen fibers respond to load. In the first region, the stress-strain curves of the hybrid materials showed a relatively low stiffness; values of the moduli E_1_ and E_2_ were lower than those of the pericardium. The fatigue tests showed that both the elongation and maximum strength of the hybrid materials are influenced by cyclic loadings, but DBP AR is less significantly affected than DBP ARLT.

**Figure 6:**
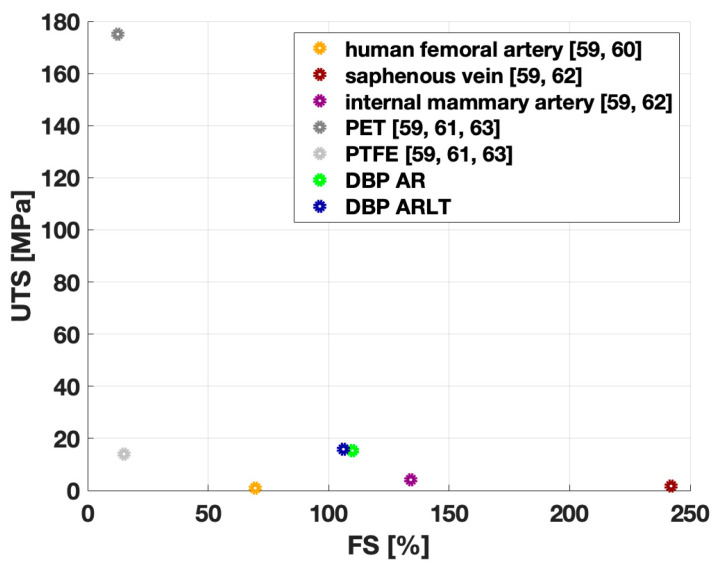
Failure strain (FS) and ultimate tensile strength (UTS) values obtained from the mechanical tests performed on the hybrid membranes DBP AR (green dot) and DBP ARLT (blue dot): they are compared with the FS and UTS values found in the literature [59,60,61,62,63] for synthetic materials (PET and PTFE) and with the autografts currently used in the clinical practice (human femoral artery, saphenous vein, and internal mammary artery).

The results obtained from the mechanical characterization of the membranes can be compared with those of materials currently used in clinical practice as vascular substitutes, both of autologous origin (e.g., human femoral artery, internal mammary artery, and saphenous vein) and synthetic origin (e.g., PET and PTFE). Figure 6 shows the UTS and FS values reported in the literature. It can be observed that the FS values of the hybrid membranes DPP AR and DPP ARLT are higher than the maximum elongation reached by synthetic materials (PET and PTFE) and the human femoral artery, but lower than the values achieved by the saphenous vein and internal mammary artery. On the other hand, the UTS values of the hybrid materials analyzed in the present study are higher than those reached by PTFE and autografts (human femoral artery, saphenous vein, and internal mammary artery), but lower than the maximum resistance value reached by PET. It is worth specifying that UTS and FS characterize the maximum capacity of the investigated materials to withstand mechanical stresses when they are experiencing non-physiological deformations. The mechanical response to the load of hybrid materials is comparable to that of human vascular tissues; therefore, they can be candidates for the replacement thereof. As regards cytotoxicity, it was assayed by a direct contact test on HYME (DBP AR and DBP ARLT). Cells were seeded directly on hybrid materials, and DBP, CF AR, and CF ARLT were used as controls. All materials promoted cell proliferation after 7 days, but the increase in metabolic activity accompanied by an increase in cell number was significant in DBP ARLT.

## 5. Conclusions

This preliminary characterization of the hybrid materials obtained by coupling decellularized bovine pericardium with synthetic polycarbonate urethanes allowed for assessing their composition and mechanical properties. The hybrid materials well adhered to each other, as demonstrated by chemical, morphological, and mechanical investigations. They also properly combine the mechanical strength of the synthetic polymers with the biocompatibility of the decellularized tissue, providing a scaffold where cell growth is favored. Therefore, hybrid materials can be intended to be promising candidates to produce vascular devices, which are able to integrate adequately with the host.

The combination of mechanical strength and impermeability afforded by the polymer, along with the biocompatibility of the decellularized tissue, positions hybrid materials as potential substitutes for the realization of cardiovascular grafts. The major advantage of hybrid materials over synthetic ones is due to their ability to be prone to re-endothelization by circulating cells. This results in increased blood compatibility, which in turn allows for reducing anticoagulation/antiaggregating therapies. This is of great relevance from the clinical point of view to avoid/mitigate the consequences of possible bleeding. Indeed, due to their versatility, other applications can be foreseen; for instance, cardiac patches and blood-contacting surfaces for mechanical circulation support devices, offering improved compatibility and durability compared to conventional synthetic or biological alternatives.

Future investigations will imply a thorough biocompatibility assessment of hybrid materials, both in vitro and in vivo. Our research group has been already engaged in a study on rats (subcutaneous implantation) to preliminarily check the absence of any adverse reaction to the presence of the implanted materials. Possible clinical translation will also require a functional evaluation in a large animal model.

## Figures and Tables

**Table 1:** Thickness values of hybrid materials. Values are expressed as mean ± SD.

Sample	CF AR	CF ARLT	DBP	DBP AR	DBP ARLT
Thickness [mm]	0.68 ± 0.46	0.93 ± 0.54	0.29 ± 0.06	0.58 ± 0.08	0.60 ± 0.12

## Data Availability

Data are contained within the article.

## References

[1] Casarin M., Todesco M., Fontanella C.G., Morlacco A., Dal Moro F., Bagno A. (2023). Hybrid Materials for Tissue Repair and Replacement: Another Frontier in Biomaterial Exploitation Focusing on Cardiovascular and Urological Fields. Processes.

[2] Chowdhury S., Pal B., Datta P. (2022). Composite Biomaterials for Bone Grafting and Other Biomedical Applications. Encyclopedia of Materials: Plastics and Polymers.

[3] Hashmi S. (2022). Encyclopedia of Materials: Plastics and Polymers.

[4] Park W., Shin H., Choi B., Rhim W.-K., Na K., Keun Han D. (2020). Advanced Hybrid Nanomaterials for Biomedical Applications. Prog. Mater. Sci..

[5] Liu X., Wu Y., Zhao X., Wang Z. (2021). Fabrication and Applications of Bioactive Chitosan-Based Organic-Inorganic Hybrid Materials: A Review. Carbohydr. Polym..

[6] Yin X., Liu Y.-Q., Zhang L.-Y., Liang D., Xu G.-K. (2024). Emergence, Pattern, and Frequency of Spontaneous Waves in Spreading Epithelial Monolayers. Nano Lett..

[7] Ghosal K., Thomas S., Kalarikkal N., Gnanamani A. (2014). Collagen Coated Electrospun Polycaprolactone (PCL) with Titanium Dioxide (TiO_2_) from an Environmentally Benign Solvent: Preliminary Physico-Chemical Studies for Skin Substitute. J. Polym. Res..

[8] Kanatani I., Kanematsu A., Inatsugu Y., Imamura M., Negoro H., Ito N., Yamamoto S., Tabata Y., Ikada Y., Ogawa O. (2007). Fabrication of an Optimal Urethral Graft Using Collagen-Sponge Tubes Reinforced with Copoly(L-Lactide/ε-Caprolactone) Fabric. Tissue Eng..

[9] Engelhardt E.-M., Micol L.A., Houis S., Wurm F.M., Hilborn J., Hubbell J.A., Frey P. (2011). A Collagen-Poly(Lactic Acid-Co-ɛ-Caprolactone) Hybrid Scaffold for Bladder Tissue Regeneration. Biomaterials.

[10] Ananta M., Aulin C.E., Hilborn J., Aibibu D., Houis S., Brown R.A., Mudera V. (2009). A Poly(Lactic Acid-Co-Caprolactone)–Collagen Hybrid for Tissue Engineering Applications. Tissue Eng. Part A.

[11] Alt E., Seliger C. (1998). Antithrombotic Stent Coatings: Hirudin/Iloprost Combination. Semin. Interv. Cardiol. SIIC.

[12] Heidenhain C., Weichert W., Schmidmaier G., Wildemann B., Hein M., Neuhaus P., Heise M. (2010). Polymer Coating of Porcine Decellularized and Cross-Linked Aortic Grafts. J. Biomed. Mater. Res. B Appl. Biomater..

[13] Stamm C., Khosravi A., Grabow N., Schmohl K., Treckmann N., Drechsel A., Nan M., Schmitz K.-P., Haubold A., Steinhoff G. (2004). Biomatrix/Polymer Composite Material for Heart Valve Tissue Engineering. Ann. Thorac. Surg..

[14] Grabow N., Schmohl K., Khosravi A., Philipp M., Scharfschwerdt M., Graf B., Stamm C., Haubold A., Schmitz K.-P., Steinhoff G. (2004). Mechanical and Structural Properties of a Novel Hybrid Heart Valve Scaffold for Tissue Engineering. Artif. Organs.

[15] Pashneh-Tala S., MacNeil S., Claeyssens F. (2016). The Tissue-Engineered Vascular Graft—Past, Present, and Future. Tissue Eng. Part B Rev..

[16] Masden D.L., Seruya M., Higgins J.P. (2012). A Systematic Review of the Outcomes of Distal Upper Extremity Bypass Surgery with Arterial and Venous Conduits. J. Hand Surg..

[17] Goldman S., Zadina K., Moritz T., Ovitt T., Sethi G., Copeland J.G., Thottapurathu L., Krasnicka B., Ellis N., Anderson R.J. (2004). Long-Term Patency of Saphenous Vein and Left Internal Mammary Artery Grafts after Coronary Artery Bypass Surgery. J. Am. Coll. Cardiol..

[18] Chang Z., Zhang J., Liu Y., Gao H., Xu G.-K. (2023). New Mechanical Markers for Tracking the Progression of Myocardial Infarction. Nano Lett..

[19] Harskamp R.E., Lopes R.D., Baisden C.E., de Winter R.J., Alexander J.H. (2013). Saphenous Vein Graft Failure After Coronary Artery Bypass Surgery: Pathophysiology, Management, and Future Directions. Ann. Surg..

[20] Klinkert P., Post P.N., Breslau P.J., van Bockel J.H. (2004). Saphenous Vein Versus PTFE for Above-Knee Femoropopliteal Bypass. A Review of the Literature. Eur. J. Vasc. Endovasc. Surg..

[21] Rocco K.A., Maxfield M.W., Best C.A., Dean E.W., Breuer C.K. (2014). In Vivo Applications of Electrospun Tissue-Engineered Vascular Grafts: A Review. Tissue Eng.-Part B Rev..

[22] Haruguchi H., Teraoka S. (2003). Intimal Hyperplasia and Hemodynamic Factors in Arterial Bypass and Arteriovenous Grafts: A Review. J. Artif. Organs.

[23] Sarkar S., Salacinski H.J., Hamilton G., Seifalian A.M. (2006). The Mechanical Properties of Infrainguinal Vascular Bypass Grafts: Their Role in Influencing Patency. Eur. J. Vasc. Endovasc. Surg..

[24] Greenwald S.E., Berry C.L. (2000). Improving Vascular Grafts: The Importance of Mechanical and Haemodynamic Properties. J. Pathol..

[25] Davies M.G., Hagen P.-O. (1995). Pathophysiology of Vein Graft Failure: A Review. Eur. J. Vasc. Endovasc. Surg..

[26] Hibino N., McGillicuddy E., Matsumura G., Ichihara Y., Naito Y., Breuer C., Shinoka T. (2010). Late-Term Results of Tissue-Engineered Vascular Grafts in Humans. J. Thorac. Cardiovasc. Surg..

[27] Haisch A., Loch A., David J., Pruß A., Hansen R., Sittinger M. (2000). Preparation of a Pure Autologous Biodegradable Fibrin Matrix for Tissue Engineering. Med. Biol. Eng. Comput..

[28] Reed A.M., Potter J., Szycher M. (1994). A Solution Grade Biostable Polyurethane Elastomer: ChronoFlex^®^ AR. J. Biomater. Appl..

[29] Todesco M., Zardin C., Iop L., Palmosi T., Capaldo P., Romanato F., Gerosa G., Bagno A. (2021). Hybrid Membranes for the Production of Blood Contacting Surfaces: Physicochemical, Structural and Biomechanical Characterization. Biomater. Res..

[30] Mudigonda J., Xu D., Amedi A., Lane B.A., Corporan D., Wang V., Padala M. (2022). A Biohybrid Material with Extracellular Matrix Core and Polymeric Coating as a Cell Honing Cardiovascular Tissue Substitute. Front. Cardiovasc. Med..

[31] Zheng Z., Eglin D., Alini M., Richards G.R., Qin L., Lai Y. (2021). Visible Light-Induced 3D Bioprinting Technologies and Corresponding Bioink Materials for Tissue Engineering: A Review. Engineering.

[32] Eberli D., Filho L.F., Atala A., Yoo J.J. (2009). Composite Scaffolds for the Engineering of Hollow Organs and Tissues. Methods.

[33] Horst M., Madduri S., Milleret V., Sulser T., Gobet R., Eberli D. (2013). A Bilayered Hybrid Microfibrous PLGA–Acellular Matrix Scaffold for Hollow Organ Tissue Engineering. Biomaterials.

[34] Yang M., Zhang Z., Hahn C., King M.W., Guidoin R. (1999). Assessing the Resistance to Calcification of Polyurethane Membranes Used in the Manufacture of Ventricles for a Totally Implantable Artificial Heart. J. Biomed. Mater. Res..

[35] Yang M., Zhang Z., Hahn C., Laroche G., King M.W., Guidoin R. (1999). Totally Implantable Artificial Hearts and Left Ventricular Assist Devices: Selecting Impermeable Polycarbonate Urethane to Manufacture Ventricles. J. Biomed. Mater. Res..

[36] Schoen F.J., Levy R.J. (2005). Calcification of Tissue Heart Valve Substitutes: Progress Toward Understanding and Prevention. Ann. Thorac. Surg..

[37] Todesco M., Imran S.J., Fortunato T.M., Sandrin D., Borile G., Romanato F., Casarin M., Giuggioli G., Conte F., Marchesan M. (2022). A New Detergent for the Effective Decellularization of Bovine and Porcine Pericardia. Biomimetics.

[38] Cassari L., Todesco M., Zamuner A., Imran S.J., Casarin M., Sandrin D., Ródenas-Rochina J., Gomez Ribelles J.L., Romanato F., Bagno A. (2023). Covalently Grafted Peptides to Decellularized Pericardium: Modulation of Surface Density. Int. J. Mol. Sci..

[39] Bagno A., Aguiari P., Fiorese M., Iop L., Spina M., Gerosa G. (2018). Native Bovine and Porcine Pericardia Respond to Load with Additive Recruitment of Collagen Fibers: Additive Recruitment of Collagen Fibers. Artif. Organs.

[40] Todesco M., Merigliano G., Candela V., Iop L., Palmosi T., Gerosa G., Bagno A. Hybrid Membranes for Blood-Contacting Surfaces: Preliminary Characterization. Proceedings of the Seventh National Congress of Bioengineering.

[41] Fidalgo C., Iop L., Sciro M., Harder M., Mavrilas D., Korossis S., Bagno A., Palù G., Aguiari P., Gerosa G. (2018). A Sterilization Method for Decellularized Xenogeneic Cardiovascular Scaffolds. Acta Biomater..

[42] Brauner J.W., Flach C.R., Mendelsohn R. (2005). A Quantitative Reconstruction of the Amide I Contour in the IR Spectra of Globular Proteins: From Structure to Spectrum. J. Am. Chem. Soc..

[43] Kurt Oldenburg (2023). LoadSpectra. MATLAB Central File Exchange..

[44] Filippi A., Sasso E.D., Iop L., Armani A., Gintoli M., Sandri M., Gerosa G., Romanato F., Borile G. (2018). Multimodal Label-Free Ex Vivo Imaging Using a Dual-Wavelength Microscope with Axial Chromatic Aberration Compensation. J. Biomed. Opt..

[45] Schindelin J., Arganda-Carreras I., Frise E., Kaynig V., Longair M., Pietzsch T., Preibisch S., Rueden C., Saalfeld S., Schmid B. (2012). Fiji: An Open-Source Platform for Biological-Image Analysis. Nat. Methods.

[46] Borile G., Sandrin D., Filippi A., Anderson K.I., Romanato F. (2021). Label-Free Multiphoton Microscopy: Much More Than Fancy Images. Int. J. Mol. Sci..

[47] Zouhair S., Dal Sasso E., Tuladhar S.R., Fidalgo C., Vedovelli L., Filippi A., Borile G., Bagno A., Marchesan M., De Rossi G. (2020). A Comprehensive Comparison of Bovine and Porcine Decellularized Pericardia: New Insights for Surgical Applications. Biomolecules.

[48] Casarin M., Fortunato T.M., Imran S., Todesco M., Sandrin D., Borile G., Toniolo I., Marchesan M., Gerosa G., Bagno A. (2022). Porcine Small Intestinal Submucosa (SIS) as a Suitable Scaffold for the Creation of a Tissue-Engineered Urinary Conduit: Decellularization, Biomechanical and Biocompatibility Characterization Using New Approaches. Int. J. Mol. Sci..

[49] (2005). E.P. Commission Council of Europe 2.6.1. European Pharmacopeia 5.0, 2.6—Biological Tests; 2.6.1 Sterility. Eur. Pharmacopoeia.

[50] (2009). Biological Evaluation of Medical Devices; Part 5, Tests for in Vitro Cytotoxicity.

[51] Huang C.-C., Chen Y.-J., Liu H.-W. (2021). Characterization of Composite Nano-Bioscaffolds Based on Collagen and Supercritical Fluids-Assisted Decellularized Fibrous Extracellular Matrix. Polymers.

[52] Huang C.-C. (2021). Characteristics and Preparation of Designed Alginate-Based Composite Scaffold Membranes with Decellularized Fibrous Micro-Scaffold Structures from Porcine Skin. Polymers.

[53] Payne K.J., Veis A. (1988). Fourier Transform Ir Spectroscopy of Collagen and Gelatin Solutions: Deconvolution of the Amide I Band for Conformational Studies. Biopolymers.

[54] Twardowski J., Anzenbacher P., Masson M.R. (1994). Raman and IR Spectroscopy in Biology and Biochemistry.

[55] Li X., Guo Y., Ziegler K.R., Model L.S., Eghbalieh S.D.D., Brenes R.A., Kim S.T., Shu C., Dardik A. (2011). Current Usage and Future Directions for the Bovine Pericardial Patch. Ann. Vasc. Surg..

[56] Crapo P.M., Gilbert T.W., Badylak S.F. (2011). An Overview of Tissue and Whole Organ Decellularization Processes. Biomaterials.

[57] Seifu D.G., Purnama A., Mequanint K., Mantovani D. (2013). Small-Diameter Vascular Tissue Engineering. Nat. Rev. Cardiol..

[58] Chrisikou I., Orkoula M., Kontoyannis C. (2020). FT-IR/ATR Solid Film Formation: Qualitative and Quantitative Analysis of a Piperacillin-Tazobactam Formulation. Molecules.

[59] Camasão D.B., Mantovani D. (2021). The Mechanical Characterization of Blood Vessels and Their Substitutes in the Continuous Quest for Physiological-Relevant Performances. A Critical Review. Mater. Today Bio.

[60] Hasan A., Memic A., Annabi N., Hossain M., Paul A., Dokmeci M.R., Dehghani F., Khademhosseini A. (2014). Electrospun Scaffolds for Tissue Engineering of Vascular Grafts. Acta Biomater..

[61] Hasegawa M., Azuma T. (1979). Mechanical Properties of Synthetic Arterial Grafts. J. Biomech..

[62] Stekelenburg M., Rutten M.C.M., Snoeckx L.H.E.H., Baaijens F.P.T. (2009). Dynamic Straining Combined with Fibrin Gel Cell Seeding Improves Strength of Tissue-Engineered Small-Diameter Vascular Grafts. Tissue Eng. Part A.

[63] Salacinski H.J., Goldner S., Giudiceandrea A., Hamilton G., Seifalian A.M., Edwards A., Carson R.J. (2001). The Mechanical Behavior of Vascular Grafts: A Review. J. Biomater. Appl..

